# The forgotten people with thalassemia in the time of COVID-19: South Asian perspective

**DOI:** 10.1186/s13023-020-01543-0

**Published:** 2020-09-25

**Authors:** Mohammad Sorowar Hossain, Enayetur Raheem, Mahbubul H. Siddiqee

**Affiliations:** 1 Department of Emerging and Neglected Diseases (END), Biomedical Research Foundation, Dhaka, Bangladesh; 2 School of Environment and Life Sciences, Independent University, Bangladesh (IUB), Dhaka, Bangladesh; 3 Department of Mathematics and Natural Sciences (MNS), BRAC University, Dhaka, 1212 Bangladesh

**Keywords:** Thalassaemia, Bangladesh, Blood transfusion, Blood donation

## Abstract

South Asia is the hotspot of beta-thalassemia, with an estimated 200,000 patients whose lives depend on regular blood transfusion. Due to COVID-19 pandemic, many countries have adopted unprecedented lockdown to minimize the spread of transmission. Restriction of nationwide human mobility and fear of COVID-19 infection has put thalassemia patients in a life-threatening situation because of an acute shortage of blood supply. As a public health preparedness strategy during a crisis like COVID-19 pandemic, the plights of thalassemia patients should be considered. Government-sponsored community blood-banks needs to be established or coverage expanded as a safety net for the thalassemia patients in lower- and middle-income countries.

## Letter to the Editor,

South Asia has been confronting a silent epidemic of thalassemia- a life-threatening inherited hemoglobin disorder. Over 200,000 estimated thalassemia patients (45–70 million carriers) live in the Indian Subcontinent whose lives depend on regular blood transfusion with chelation therapy [1–3]. There is an acute crisis of availability of blood for thalassemia patients especially in South Asia.

While access to blood remains a persistent challenge, the coronavirus pandemic (as of April 11, 2020) has exacerbated the circumstances. To minimize the spread of SARS-CoV-2, the majority of the South-Asian countries have adopted lockdown measures. The lockdown has impacted people's lives in unprecedented ways—for example, the lack of public transportation to restrict people's movement. It is likely that such restrictions will also make it difficult, or even impossible at times, for both the blood donors and recipients (i.e., the thalassemia patients). Data do not exist to measure how this would impact the lives of thalassemia patients. Understandably, the ongoing lockdown is likely to put a significant portion of the patients into a life-threatening condition due to an acute shortage of blood supply at the community level. Community clubs and NGOs have to postpone blood donation programs. In addition, friends and relatives are not coming forward to donate blood due to panic of COVID-19 infection [4, 5]. For instance, in Kolkata (India), over 80% of blood supply in 108 blood banks comes from blood donation camps.

Uncertainty around COVID-19, followed by lockdown, may impact Bangladesh significantly. Here 10–12% of the 160 million population are thalassemia carriers, and the issue is largely neglected at the policy level [1]. A recent community-level study showed that the educated segment of the society (over 67% of the college students) have not heard about "thalassemia." Furthermore, 40% are reluctant to donate blood for thalassemia patients due to misconceptions and stigmatization [6].

Another hospital-based study found that over 62% of HbE beta-thalassemia patients are transfusion-dependent, requiring transfusion 1 to 4 times every month [1]. Most of the specialized hospitals for thalassemia patients are in Dhaka, the capital city of Bangladesh. Around 70% of the people of Bangladesh lives in rural areas. Moreover, most families cannot afford to travel to Dhaka for treatment purposes due to financial constraints; the lockdown has made the situation even worse due to the unavailability of public transports. Our unpublished report found that 78% of the families with thalassemic children struggle to get blood who could afford to come to the Dhaka city for treatment (Bangladesh Thalassemia Samity Hospital).

The current lockdown has exposed a severe weakness in the supply chain of blood, particularly for thalassemia patients. Recruiting voluntary donors is a big challenge in developing countries due to a lack of awareness. In Bangladesh, according to a WHO report, over 600,000 units of blood were collected against an estimated demand of 800,000 in 2016 [7]. It is likely that 70,000–80,000 thalassemia patients were left out of this count since thalassemia is not considered a public health problem. Only 31% of the collection is from voluntary donors while two thirds (~ 70%) come from relatives and friends of the patients [7]. Only half of the district health facilities (out of 64) have blood banks and nearly 41% of these faces a shortage of supply [7]. Moreover, a significant portion of the collected blood becomes unusable due to inadequate storage facilities, underutilization, and limited shelf-life of blood.

In future public health preparedness strategy during a crisis like COVID-19 pandemic, the issue of transfusion-dependent patients must be prioritized, particularly in thalassemia prone countries in South Asia. In this perspective, the community-based blood banking needs to be encouraged and expanded with government sponsorship to keep the vulnerable patients within a safety net.

## Data Availability

Not applicable.
